# Bioremediation of Organic Pollutants in Soil–Water System: A Review

**DOI:** 10.3390/biotech12020036

**Published:** 2023-05-11

**Authors:** Pankaj Kumar Gupta, Manvi Gandhi

**Affiliations:** 1 Faculty of Environment, University of Waterloo, Waterloo, ON N2L 3G1, Canada; 2 Faculty of Sciences, University of Adelaide College, Adelaide, SA 5000, Australia; manvi.gandhi@adelaide.edu.au

**Keywords:** NAPL, BTEX, soils, pollution, groundwater, bioremediation

## Abstract

Soil–water pollution is of serious concern worldwide. There is a public outcry against the continually rising problems of pollution to ensure the safest and healthiest subsurface environment for living beings. A variety of organic pollutants causes serious soil–water pollution, toxicity and, therefore, the removal of a wide range of organic pollutants from contaminated matrix through the biological process rather than physico-chemical methods is an urgent need to protect the environment and public health. Being an ecofriendly technology, bioremediation can solve the problems of soil–water pollution due to hydrocarbons as it is a low-cost and self-driven process that utilises microorganisms and plants or their enzymes to degrade and detoxify pollutants and thus, promote sustainable development. This paper describes the updates on the bioremediation and phytoremediation techniques which have been recently developed and demonstrated at the plot-scale. Further, this paper provides details of wetland-based treatment of BTEX contaminated soils and water. The knowledge acquired in our study contributes extensively towards understanding the impact of dynamic subsurface conditions on engineered bioremediation techniques.

## 1. Introduction

**Figure 1:**
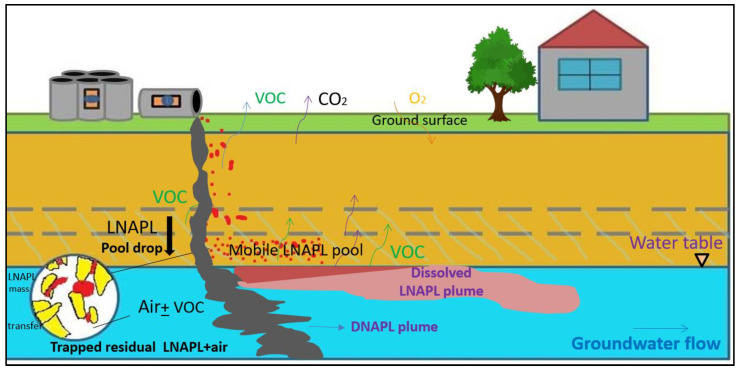
Flow of organic contaminants in soil–water systems under varying groundwater flow conditions, adapted from Gupta et al. [5].

Hydrocarbons such as benzene, xylene and other BTEX compounds can exist in a soil matrix as free phase, generally referred to as non-aqueous phase liquids (NAPL), and as dissolved or gas phase. Considering their viscosity, NAPLs have been classified as light non-aqueous phase liquids (LNAPLs) and dense non-aqueous phase liquids (DNAPLs) [1,2,3]. As, NAPLs are immiscible with water, hence even if small quantity of NAPL got introduced into the soil, it can create a plume in the subsurface system through vertically downward flow, which is enough to degrade the soil’s quality (Figure 1) [2,4]. Later, it also reaches the groundwater table where it contaminates the groundwater, which is used for different purposes such as irrigation and drinking water supply, on a large scale. The small quantity of NAPL which got dissolved in the water is enough to create cardiovascular, neurological, and various other maladies in humans and impose health hazards to the terrestrial as well as aquatic inhabitants For example, it can cause chronic ailments such as cancer and has been found to affect the homeostasis of the human body, which in turn gives birth to various lifelong diseases. Hence, understanding the transient flow of NAPLs in the subsurface heterogeneities is the precursor to approaching remedial actions. Therefore, this paper presents an in-depth study on field-scale behaviour, laboratory studies and analytical and numerical modelling in multiphase flow to understand immiscible fluid behaviour and characterize remedial actions [1,2,3,4].

## 2. DNAPL in Soil–Water System

This section introduces a synoptic view of DNAPL contamination accompanied by a comparative analysis between preceding and current trends employed to predict DNAPL contamination in the heterogeneous aquifer system. There is an excessive use of dense non-aqueous phase liquids in industries, such as chlorobenzenes, chloromethanes, PCBs, tetra and trichloroethene, creosote and coal tar, which are immiscible with water and often leave residual content in the subsurface environment; this has been reported by Parker et al. [6], Rasa et al. [7] and Yang et al. [8]. The accumulation of residual DNAPL contaminants in the complex heterogeneous system, such as in LPPM (low-permeability porous media), and storage of contaminants in the sorbed and dissolved phases are reported in various studies. These commonly found contaminants in the subsurface environment have posed a malignant life threat to human health and the whole ecosystem. Therefore, understanding the fate and anomalous transport behaviour of DNAPL and factors and processes affecting the plume evolution are a must to investigate prior to any field-scale remedial action plan, and so this study brings out and unpack all the major studies through bibliometric analysis to look into and assess the aquifer vulnerability. This study also extends the knowledge on the influence of heterogeneity and distance time dependency of dispersion for understanding the plume evolution.

DNAPL-contaminated sites have been reported in several countries, including the USA, Canada and Australia, many other countries in Europe and Asia. In the USA alone, there are estimated to be over 14,000 DNAPL-contaminated sites. Many of these sites are associated with historical industrial activities and military operations. In Europe, the largest numbers of DNAPL-contaminated sites are found in Belgium, Germany, and the Netherlands, where there is a legacy of industrial pollution. In Asia, DNAPL contamination is a significant problem in countries such as China, Japan, and South Korea, where there has been rapid industrialization and urbanization [9]. Ering et al. [10] found the impact of high groundwater velocities on the position, architecture and geometry of TCE. Further, they suggested that notwithstanding this, the study was restrained to lab-scale conditions only. Eventually, a strong correlation between TCE concentration and hydraulic head was observed near the source transect. However, this influence was weakened in the locations away from the recharge area [8]. Some studies were limited to homogenous layers with the neglected impact of LPPM (low-permeability porous media) on transport behaviour. Observations in this regard suggest the need for clearer investigation of heterogeneity and fluctuating groundwater table conditions. DNAPL plume insistence in the heterogeneous porous system relies upon forward and backward diffusion through an LPPM/stagnant region within the aquifer, hence understanding the mass transfer between LPPM and aquifer is a must. Guo and Brusseau [11] in particular reported that a large heterogenous porous system was found to be associated mainly with mass transfer constraints from the stagnant zones. Further, they observed that non-ideal mass removal behaviour was observed even for the homogeneous system, which can be solely attributed to the good field configuration and mass transfer constraints and back diffusion from LPPM in the layered or highly heterogeneous system. Moreover, it has been observed that contaminant mass transfer between mobile and stagnant regions depends upon internal hydraulic gradients between low- and high-permeability materials.

A weak negative correlation between aquitard thickness and D* was observed. Processes such as dissolution, sorption–desorption in the mobile aquifer and stagnant region, degradation and transverse mixing were analysed. It was found that DNAPL dissolution, injection flow rate, distance to observation wells and hydrogeological heterogeneities were the predominant factors governing the travel time of dissolved contaminant. Yang et al. [8] observed that plume persistence near the source zone was affected by non-ideal sorption following DNAPL depletion. Large variations in the plume stupefaction were observed for various sorption models, indicating the need for the selection of a sorption model for the prediction of plume longevity. Moreover, non-uniform degradation rates can simulate the contaminant transport at actual field-scale conditions efficiently as compared to the conventional constant degradation rate scenario [12]. The comparison among α longitudinal, α transverse, α lateral was also performed to analyse the variation of dispersity, and the noticeable gap between dispersity values along different directions was unveiled, indicating the importance of high contrast in the dispersity values used in the modelling. This suggests the further need to emphasize transverse dispersivity in the simulation of DNAPL transport through a high aquifer system.

Several modelling frameworks were developed for different purposes, such as an aquitard diffusion model, based on source zone dissolution and diffusive transport to study the effects of DNAPL source architecture and back-diffusive fluxes from LPPM regions. Further, an SFDM (single fissure diffusion model), multi-tracer approach and SFDM modelling were employed for the required investigation [13]. Yang et al. [8] developed a 1D analytical solution to analyse the effect of exponential source depletion and back diffusion from LPPM on long tails in the contaminant BTCs. A semi-analytical approach, namely, FT-MIKSS was developed for the approximation of thedissolved-phase plumes of BTEX in a heterogeneous aquifer [14]. Further, it was found that FT-MIKSS was 100–1000 times computationally faster than numerical models (RT3D, PHT3D, PHAST) for tested examples. An integrated contaminant elution and tracer test toolkit (ICET) was developed to improve characterization of mass removal behaviour and to represent the mass transfer processes that can impact the DNAPLs’ transport in the complex subsurface environment [15]. The conclusion suggests that a hybrid analytical–numerical modelling approach should be implemented, meanwhile simulating the transport phenomena of DNAPL at field-scale conditions, as anomalous transport behaviour of DNAPLs in the aquifer system has always been the subject of numerous modelling studies [7,11,12,16]. Applicability of numerical models such as HydroGeosphere, MODFLOW/MT3DMS and FEELOW to simulate the diffusion into and out of an LPPM in a sandy aquifer system were examined [17]. Bianchi and Zheng [18] reported the use of a lithofacies-based approach with a 3D stochastic numerical model to simulate DNAPL transport behaviour at the macro-dispersion experiment (MADE) site in Mississippi, USA.

A review of these studies suggests the need for numerical-modelling-based field-scale studies considering unknown or unidentified parameters such as dispersion coefficients and the mass transfer coefficient computation by coupling the contaminant model with an inverse optimization algorithm. A summary of research works on organic pollutants in a soil–water system has been listed in Table 1. Further research gaps need to be bridged considering DNAPL contamination in the heterogeneous system. For instance, variable nature of flow and transport parameters can be considered in 2D and 3D lab simulations. Further spatial and temporal aspects, microscale flow and transport processes in stagnant or mobile regions and through LPPM should be considered or surveilled vigilantly. Considering the various unidentified flows and factors affecting the DNAPL transport behaviour, field-scale implementation must be checked, understanding the various limitations of the controlled lab experiment.

## 3. LNAPL in a Soil–Water System

Toxic or carcinogenic discharge of LNAPL hydrocarbons in the soil and water in coastline areas has been rampantly contaminating the semiarid landscape in coastal proximity, inducing substantial pitfalls and damage to aquatic life as well as settled populations in the vicinity [1,2,3,5]. The complication is more perilous in regions where petroleum and refinery industries are based [1]. This paper explores the pluses and minuses of bioremediation of LNAPLs in coastal (semi)arid environments under disparate environmental factors that affect the implementation of bioremediation. These factors include oxygen supply, soil moisture, pH, nutrients, temperature and redox potentials, the properties of targeted pollutants and site properties, etc., among which, soil moisture, temperature variation and the shifting groundwater table dynamics are considered to play pivotal roles and set optimal conditions in biodegradation expediency of the polluted semiarid and arid coastland [1]. Soil moisture predominantly affects the fate and transport of LNAPL in polluted sites [19], which in succession influences the total water potential impeding oxygen supply as well as the aqueous phase nutrients to microbiota through its correlation to water film thickness [20]. Shortening soil moisture also leads to the nonvoluntary death of microorganisms as a result of metabolic dysfunction and desiccation (JRB). Moreover, it has been observed that microbial degradation rates are extreme at a soil–water potential between 0 and −1 bar [21], as the applicability of biodegradation through soil–water content is compound and soil-specific; consequently, it has been observed that degradation of aryl hydrocarbons is extremely limited to the soil moisture parameter. It was found that degradation is greater at 80% than at 40% of soil field capacity; optimum water content of field capacity for speedy biodegradation of aryl hydrocarbon was found to be between 50 and 70%, and since low chain aliphatic hydrocarbons have low solubility, their mineralization is greatly affected by soil–water content. A comprehensive examination is indispensable to extrapolate the impact of different environmental factors such as soil characteristics and salinity, water table dynamics, in addition to diurnal and seasonal inconstancy of temperature in semiarid coastal regions. These factors emphatically alter LNAPLs’ properties, rates of biodegradation, mass transfer rates and activity or survival of degraders. Because temperature controls the metabolic function of microbes, degradation was intensified with increasing soil temperature, and observation suggests that different soil microorganisms, such as psychrofiles, mesophiles and thermophiles, grow at varying temperatures, among which mesophiles were found to be extremely active in the temperature range of 20–35 °C and were also major degraders of hydrocarbon. A mixture of thermophilic aerobic bacteria was successfully implemented to degrade diverse components of LNAPLs containing wastewater at a constant temperature of 40–42 °C; however, customizing the soil temperature in the field is a tough task.

The microbial degradation rate is also regulated water table dynamics [2]. As, the LNAPLs move downward and the water level goes down, it leaves residual fraction in the vadose zone in the form of isolated ganglia [2,3]. Further, as the water level goes up, LNAPLs too are shifted to the upward zone, leading to the entrapment of LNAPLs and air by a snap-off or bypassing mechanism in the smear zone below the groundwater table [2]. The fate and transport of the accumulated LNAPL pool and corresponding intermittent ganglia/blobs in the smear zones are important considerations in bioremediation of polluted sites owing to additional chemical and hydraulic heterogeneity in space and time introduced by the fluctuating water table. Sinke et al. [22] examined the impact of water table dynamics on redox conditions and transport of dissolved toluene and 4-nitrobenzoate in a sand column, and significant differences in contaminant transport and redox conditions were analyzed. After all it is quite evident that the fluctuating the microbial degradation rate, mass transfer and transport of soil air substrate throughout the variably saturated zones. The combined impact of the availability of soil moisture, soil temperature inconstancy followed by fluctuating water table dynamics with a mass transfer is likely to result in the wide application of biodegradation. Furthermore, soil heterogeneity of polluted sites will improve the findings of LNAPLs’ fate and transport under dynamic water table condition, as the environmental factors relevant to arid and semiarid coastal regions are interdependently corelated. Hence bioremediation must be performed under the integrated, varying underlying environmental factors.

## 4. Mobility of NAPL in the Experimental Domains

Gupta et al. [2] performed a series of two-dimensional experiments to investigate the fate and transport of light NAPL in mineral aquifers under dynamic groundwater table conditions by incorporating the soil water from the Panipat refinery area. They provide strong experimental and numerical evidence that groundwater fluctuations play a significant role in (1) spreading of the pure phase NAPL in the capillary zone, (2) accelerating dissolution from a large pool area, (3) dissolved NAPL movements and (4) the biodegradation of dissolved plumes having different initial concentrations. Likewise, Gupta and Yadav [3] designed a three-dimensional experimental sandy aquifer and performed a series of tests under varying groundwater flow conditions. They reported the average mass transfer coefficient at different groundwater velocities, which increased proportionally with velocity toward a limiting value. Parameter estimates for the mass transfer coefficient, Sherwood number and Peclet number were determined at different hydrodynamic conditions. They found a large-dissolved plume under high groundwater velocity, which create multi-concentration profiles. Biodegradation rates were high in regions having dissolved NAPL concentrations between 50 and 100 ppm. Lower biodegradation rates were observed for lower (<50 ppm) and higher (>100 ppm) concentrations of dissolved NAPL, which signifies the dependency on initial dissolved NAPL concentrations.

## 5. Fate of BTEX under Seasonal and Diurnal Fluctuations of Soil–Water Temperature

The present study [1] monitors the potency of biodegradation on monoaromatic hydrocarbons and BTEX compounds under differing soil–water temperatures, as among other environmental parameters, including high/low pH, water table dynamics and varying soil moisture content, etc., soil–water temperature is a critical parameters to manipulate in bioremediationprocedure [23]. It is also anticipated that fluctuating soil–water temperature may impact properties of hydrocarbon, rate of degradation, rate of mass transfer and microbial activity of degraders; microbial metabolism is also boosted with intensifying soil temperature. To examine all these factors, batch experiments were performed at three different persistent temperatures as well as at another set of inconsistent temperatures to simulate diurnal and seasonal fluctuations. Set up on calefaction, different soil microorganisms such as psychrophiles, mesophiles and thermophiles were employed to examine the rate of biodegradation; mesophiles were found to degrade most hydrocarbon in the temperature range of 20–35 °C [24]. Moreover, this range can trigger a degradation rate of twice or thrice owing to a 10 °C increase in temperature, whereas too much of a rise in temperature also impedes the rate of degradation. Similarly, thermophilic bacteria were found in the soil temperature of 50 °C in the Kuwaiti desert, which reveals the efficacity of bioremediation in hot soil–water environments, and it has been observed that psychrophiles degrade pollutants in cold regions. In the controlled lab experiment, the first set of batch experiments was performed following three consistent soil–water temperature conditions; 21 °C (room temperature), 10 °C (low temperature) and 30 °C (high temperature) were determined to act as temperatures similar to the spring /autumn, winter and summer season. A noticeable amount of biodegradation (98.4%) of toluene was found in the first two days of the experiment [1]. The overall result of this experiment shows that the rate of degradation of toluene was increased twofold for every 10 °C rise in temperature [1]. For the second set of the batch experiment under fluctuating soil–water temperatures, it took 70–75 h for the toluene to degrade in the temperature range ranging between 21–10 °C; to simulate diurnal variations which occur in the warm weather, the temperature range of 21–30 °C was taken into consideration. The aftermath of this experiment shows that it took 35–45 h for the toluene to degrade; moreover, for the extreme case of 10 °C and 30 °C, 45–55 h were required for the degradation of the same amount of toluene. Overall, the results of this batch experiment show that degradation time for fluctuating scenarios is comparatively longer. However, a diurnal differential in temperature along with other environmental factors of a specific site must be taken into consideration prior to experimentation. To date, only a limited number of studies have explored the use of inverse optimization algorithms to combine pollution models with uncertain parameters such as dispersion coefficients and mass transfer coefficients for field studies and numerical simulations. Certain bacteria, such as Pseudomonas, Rhodococcus and Mycobacterium, have been found to be effective in breaking down BTEX compounds. The specific type of bacteria involved in the degradation process can depend on factors such as the availability of oxygen and other nutrients, pH, temperature and the presence of other contaminants [2,3,11,16].

**Table 1:** Summary of the research literature on the presence of organic contaminants in soil–water systems.

Reference	Contaminants	Techniques	Remarks
adapted from [25]	(1) 40 mL vials 10 g soil (2) 250 mL microcosm with 20 g soil	(1) Soil–water content was (i) 100%–50%, (ii) 20% (2) Arid volcanic indigenous population	(1) 0.03 mg (L h)^−1^ (2) 1–7 × 106 mg (gODsoil h)^−1^
adapted from [26]	63 mL microcosm with no headspace	31% soil–water content and indigenous microbial population	0.12 h^−1^
adapted from [27]	120 mL of mixed gas phase benzene in a microcosm slurry	50–100% soil–water content and indigenous from contaminated site at different depths	0.21 h^−1^
adapted from [28]	14.8 L biofilm membrane reactor	Matric potential from 0 to −1.5 MPa and adapted culture of P. putida biofilms	18–55 mg toluene (mg protein h)^−1^
adapted from [29]	Bioreactor D: 3.8 L: 30.48	Soil–water content 14% to 20.5% and contaminated site	0.11 mg (L h)^−1^
adapted from [30]	Unsaturated column D: 25 L: 20–30	Soil–water content 15%–12%–8% and indigenous microbial population	1.42 h^−1^
adapted from [31]	Unsaturated column infiltration D: 10 L:170	Soil–water content 65% and indigenous microbial population	0.33–1.46 mg (kg h)^−1^
adapted from [32]	Underground petroleum storage leaks	Surface flow	Benzene: 48%
adapted from [33]	Refinery wastewater	VSSF VSSF CWs	BOD5: 68–70% COD: 63–65% NH4+-N: 49–68% NO3-N: 54–58% PO4 3 + P: 42–42% remove
adapted from [34]	Refinery wastewater	Vertical-flow soil filter systems—rough filter (RF)	MTBE: 70% benzene: 98% remove

## 6. Remediation of NAPL-Contaminated Groundwater Using Plant

This study explores the redressal of BTEX-compounds-contaminated groundwater employing a conjoint expedient procedure of bioremediation, taking multifaceted aspects into consideration such as cost-effectiveness, feasibility on a macroscale and efficacy of the techniques under different environmental factors. For experimentation, polluted groundwater was fetched from Panipat oil refinery located in Haryana, India, for biodegradation, applying plant-assisted biostimulation and bioaugmentation techniques [35]. Four different bioremediation techniques were analogously employed to investigate the rate of degradation, prolongation of lag phases, as well as time span of degradation [35]. In the first two cases, natural biodegradation and biostimulation were found; in the third case, discernibly, the plant-assisted biostimulation was found more efficacious. In the fourth scenario, having fused with bioaugmentation, the efficacy of decontamination was advantageous among the aforesaid two techniques [35]. Plant-assisted biostimulation and bioaugmentation performed in a controlled environment at room temperature (21 °C sustaining the initial substrate concentration as 8 mg/L), exhibited the maximum rate of degradation with accrual of 97.93% juxtaposition to natural biodegradation separately. Locally available wetland plants of Canna generalis were utilised to increase the removal efficiency of the LNAPL, as the root exudates play a dominant role in the biostimulation of the rhizosphere, providing a benign carbon source for toluene degraders [4,35]. The batch experiment showed the decay of toluene in every phase, whereas sterile batches demonstrated comparatively small losses of the LNAPL compared to biodegradation assays over the whole experimental period. The coadjuvant effect of biostimulation and plant-assisted degradation raised the degradation rate to 44.85% in comparison to natural biological attenuation and 47.8% in comparison to biostimulation separately, as rhizospheric microbiota, after having been catalysed by root exudates are proven more effective than the indigenous microbial population in a bioremediation scenario [36]. The exiguous lag phase, even in that condition when nutrient availability is the same as it is in the plant-assisted bioremediation techniques, prioritizes the noteworthiness of the rhizosphere in expeditious acclimatization of microorganisms for remediation of a LNAPL-contaminated soil–water system. McFarlane et al. [37] examined the rhizosphere and found that it can act as a flawless sink for the depletion of benzene vapours; moreover supporting this synergistic approach, it has been shown through experiments that C. generalis plants are capable of growing even though a high concentration of the LNAPL is sustained Over the period of five weeks; however, plants controlled the infiltration of contaminants into the groundwater. Still, transversal dynamics of LNAPL and the depth of contamination in the aquifer system and site-specificity must be taken into account for optimizing the efficacy. In the phytoremediation process, plants can help to fix the soil and reduce NAPL infiltration into groundwater [Table 2].

## 7. Pot-Scale Wetland Treatment of BTEX

Percolation of BTEX compounds into the groundwater table is a perilous concern due to their lethal toxicity, so it is indispensable to seek and examine better remediation options that are environmentally nonfatal; among all other methods of remediation, such as physical containment, booming and skimming, soil–water extraction and a host of others, this paper [4] examines the removal efficacy of plant-assisted engineered bioremediation techniques using pot-scale wetlands to quantify toluene absorption by root and shoot biomass. Dzantor [38] observed that plants may create a suitable environment for the metabolism of microorganisms, in turn intensifying the rate of biodegradation in the contaminated root zone, as the phytoremediation of organic contaminants transpires sharply via root uptake and eventual translocation to shoot biomass, phytodegradation and rhizodegradation. Plant root exudates act as an adjuvanted substrate to excite microbial activities in the root zone. To verify the aforesaid experiment, the *Canna generalis* plants were grown in sets of two mesocosms, named M1 and M2, using primary treated domestic wastewater as a growth media in all mesocosms [4]. One mesocosm, M1, was contaminated with 150 mg/L toluene to accommodate the rhizospheric microbes with pollutant; in the second mesocosm, M2, the plant shoot biomass was cut out for excluding the loss of transpiration from the xylem. Loss of toluene and water were quantified diurnally after spiking both the mesocosms with an inchoate toluene molality of 120 mg/L. Later, the removal of toluene from the rhizosphere pore water of the planted and unplanted mesocosms was simulated using a set of mass balance equations in order to include aqueous diffusion of toluene towards the root surface and its eventual translocation from root to shoot biomass. Observation showed that toluene was degraded in all three mesocosms, but the rate constant of toluene removal was higher for planted mesocosms with shoots (MI) in contrast with mesocosms M2 and M3, which can be assigned to the function of plants in wetlands [39]. Even plant-assisted bioremediation reduced the time required for biodegradation by up to 25%. An accelerated biodegradation rate was attained by conjoining biostimulation and bioaugmentation techniques of engineered bioremediation with phytoremediation in wetland mesocosms. Results showed that the mass toluene that accumulated in the root biomass was greater compared to shoot biomass. In contrast with the controlled simulation, field scenarios may show a little diffraction due to other indigenous microbiota present and diverse fluctuating environmental factors; notwithstanding this, plant-assisted bioremediation is preferable for hydrocarbon-polluted lands [Table 2].

## 8. Case Study of Duplex-Constructed Wetlands for the Treatment of Diesel-Contaminated Wastewater

**Figure 2:**
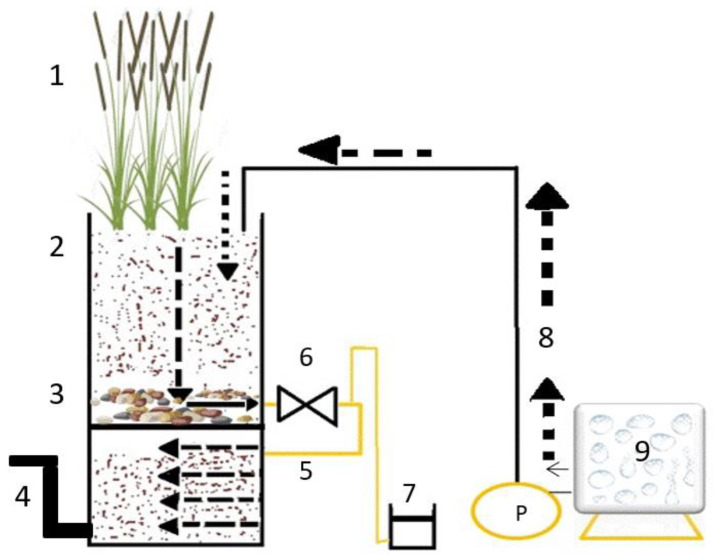
Systematic diagram of duplex wetland used to remediate organic pollutants from wastewater, adapted from Mustapha et al. [40]. 1—Phragmites australis, 2—sand, 3—gravel, 4—outlet pipe, 5—pipe connecting the compartments, 6—valve, 7—effluent collection bucket, 8—inlet pipe and 9—influent tank.

The present study [40] looks over the qualitative application of duplex CW (constructed wetlands) in the removal efficacy of wastewater contaminated with diesel compounds fluctuating from C7-C40 and heterogeneous BTEX compounds, which spawn lethal health complications for aquatic as well as terrestrial life. The hybrid constructed wetland is a conjunction of two or more back-to-back connected constructed wetlands, which conjoin the functionality of single constructed wetlands to furnish rectified effluent water quality (Figure 2). A duplex CW is a hybrid constructed wetland composed of a VF (vertical flow) CW on top of an HF (horizontal flow) CW, with extra features of a reduced space requirement [40]. Saeed and Sun [41] examined how the integrati on of vertical flow (VF) and horizontal flow (HF) conjointly enhance organic and nitrogen removal efficiency due to the presence of aerobic, anaerobic and anoxic phases. Dittmer et al. [42] observed that CW2D is capable of modelling the role of organic matter, nitrogen and phosphorus in the biochemical elimination and transformation processes of organic pollutants. The present experiment was simulated using the same CW2D to inquire into the degradation of target pollutants. The three duplexes, namely, CW1, CW2 and CW3, were spiked with different concentrations of nutrients for the removal of organic contaminants, employing a series of practical and numerical experiments. Placed in a climate-controlled greenhouse, the duplex-CWS has two parts; the first one is a vertical flow CW planted with *Phragmites australis* and the second one is an unplanted horizontal flow filter (HFF). The nutrient level was customized after two weeks by adding different concentrations of mineral nitrogen and phosphate to each of the influent tanks INF1, INF2 and INF3, corresponding to the concentration of 10, 30 and 60 mg/L, respectively, of ${NH}_{+4}^{-}N$ and 3, 6 and 12 mg/L, respectively, of ${PO}_{4}^{-3}$ P between 21 and 56 days. Numerical experiments depicted that a reduction in PH of the effluents would be found. Furthermore, it was quantified that C7-C40 alkane compounds were reduced by more than 90% from the three duplex-CWs, and that the duplex-CWs exhibited more enhanced removal efficiencies of DRO, toluene, ethylbenzene, xylene and O-xylene than did the VF CWs. Avila et al. [14] found that 50–100% removal efficiency may be subject to biodegradation, sorption, hydrolysis and photodegradation processes within the hybrid duplex-CWs. The VF CWs excellently reduced ammonium concentration in all three duplex-CWs as compared to the very low (6–29%) rate in the three HFF compartments. All these findings suggest that the duplex-CW has a higher removal efficiency of petroleum contaminants; in addition, the hybrid CW (VF CW + HFF) showed much more efficient removal efficiency due to time duration, size and land requirements, and the role of the plant must be taken into consideration. In field observation, it is exigent due to fluctuating aspects such as temperature, etc.

## 9. Integrated Column and Wetland Study

**Figure 3:**
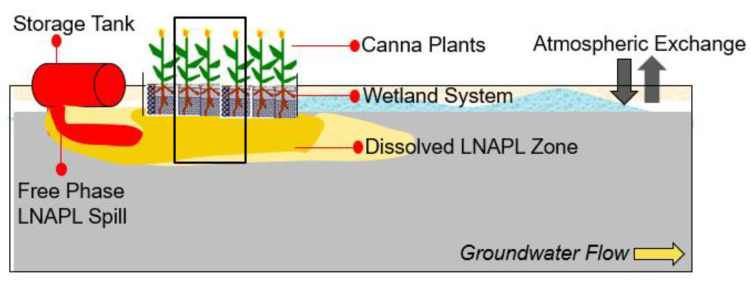
Systematic diagram of constructed wetland used to remediate organic pollutants from wastewater, adapted from Basu et al. [4].

The present study [4] outlines the on-spot redressal of LNAPLs and examines the nuts and bolts of the complications that result due to its release at the surface and ensuing migration to the downgradient location through hydrodynamic dispersion, especially toluene in the vadose zone, by simulating integrated vertical soil column and wetland. For several reasons, petrochemical discharge has been continuously a grave challenge due to its lethality when it infiltrates into the groundwater system [4]. Among all other prevailing remediation techniques, such as skimming, pump treat, air sparing, etc., bioremediation is the most promising technique because it is safer and a pioneering novel technique. In order to examine the efficacy of this technique, a wetland-assisted integrated soil colums was designed for in-situ bioremediation was carried out following numerical modelling and experimental setup using a vertical soil column and wetland (Figure 3). Tindall et al. [43] and Ranier et al. [44] have jointly assessed the performance of pollutant removal either by sand columns or in wetlands. To simulate the actual field scenario of the contaminated vadose zone, a laboratory experiment was carried out using a large vertical column setup of 120 cm high plexiglass with an inner diameter of 15 cm, with 23 sampling ports fixed at an equal spacing of 5 cm along the column depth. The homogeneously packed clean aquifer material’s (particle size of 0.5–1.0 mm) porous medium was oversaturated prior to filling into the column setup to create a homogeneous packing. Polluted groundwater was collected and toluene-containing water from the carboy was mixed with wetland water; this was then applied as a continuous source of 250 pm toluene flux to the attached column using a peristaltic pump, maintaining a constant groundwater velocity of 0.625 cm h^−1^ in the vertical direction throughout the experiment. The column setup with planted wetland was aimed to evaluate the toluene biodegradability of the *canna generalis* plant, and another setup without the plant was used for comparative evaluation. Transport of toluene in a variably saturated soil profile was expressed by the advection–dispersion equation. A breakthrough curve (BIC) shows that the relative concentration of toluene reached up to a breakthrough level, which was 0.5 in =60 h at the lowermost port at 85 cm depth. Notwithstanding this, the experimental time to reach 0.5 relative concentrations was only =40 h in the unplanted wetland for the same sampling port; this clearly shows that plants affect the attenuation of toluene predominantly. A variation in the relative concentration of toluene between input and output fluxes at 100 h was found as 13.34% for unplanted and 30.86% for the planted setups. This also shows that planted setups have enhanced biodegradation by a factor of 2.5, in comparison to unplanted wetlands. All these findings point towards the fact that plant-assisted engineered bioremediation techniques enhance the biodegradable efficacy of a microbial population, indicating the favourable role of the rhizospheric zone. The BCs of the selected pollutant were also simulated using flow and transport parameters through preliminary batch and column experiments. Findings on engineered bioremediation techniques can be very useful for treating the pollutant in a less disruptive and more fruitful way [Table 2].

## 10. Engineered Bioremediation of NAPL-Polluted Sites

Given that the ultimate goal of remediation practices at contaminated sites is to return the entire subsurface to its uncontaminated condition at a minimum cost and time, a low-cost bioremediation system design is urgently needed. The impact of soil moisture content and groundwater temperature on NAPL biodegradation was previously studied; however, attention to their combined effect was not considered. Gupta et al. [5] designed a unique bioremediation system by incorporating practical experiments, process-based models and optimization simulators to remediate NAPL-polluted sediments under varying soil moisture and temperature conditions. Their study considers their combined effect on the biodegradation of dissolved toluene through a series of microcosm experiments. They found the biodegradation rates were high at 30 °C for batches having soil moisture conditions near field capacity, as compared to colder conditions with similar moisture levels. The degradation rates were reduced considerably at low temperatures at a low soil moisture content. The remediation time was also found to be significantly less for a high moisture–high temperature combination. They suggest that the application of in situ bioremediation methods in the region of high temperature, with moisture content near field capacity, is the most efficient and cost-effective. Gupta et al. [5] provide a unique engineered bioremediation design for treating hydrocarbon-polluted variably saturated zones in different seasons.

**Table 2:** The research literature examines the effects of treatment approaches on organic contaminants removal in soil–water systems.

Reference	Contaminants	Techniques	Remarks	Removal
adapted from [45]	Sandy beach with light crude oil in it.	Bioaugmentation and biostimulation using microbial inoculation or inorganic mineral supplements.	Unsuccessful.	
adapted from [46]	Simulation of an oil spill (light crude and fuel oil) at mature mangroves.	Forced aeration and food addition for biostimulation.	Successful.	No apparent reduction in mortality of trees with bioremediation.
adapted from [47]	Degradation of crude oil in harsh sub-Antarctic conditions.	Slow-release fertiliser Inipol EAP-22 and three different fish composts for biostimulation.	Successful.	95% removal.
adapted from [48]	Oil deterioration on a salt marsh at the coast.	Fertilisation with N and P for biostimulation.	Successful.	GC/MS resolved alkanes and aromatics degraded substantially by >90% and >80% respectively.
adapted from [49]	Degradation of petroleum hydrocarbons in the polar desert.	Fertilisers (urea and diammonium phosphate) and surfactants are used in biostimulation together with temperature and moisture content modifications.	Successful.	In the laboratory, significant removal of compounds > nC16 occurred, whereas in the field, TPH reduction was mainly limited to removal of compounds < nC16.
adapted from [50]	Soil contaminated with total petroleum hydrocarbons (TPH) and PAHs from oily sludge.	Biostimulation using manure.	Successful.	After 1 year of bioremediation TPH in the treated plot had decreased by 58.2% compared with 15.6% in the control plot.
adapted from [51]	Severe deterioration of crude oil.	Using commercial microbial culture for bioaugmentation.	Successful.	
adapted from [52]	Benzene.	Vertical flow CW.	Successful.	Removal efficiencies between 88.71% and 89.77%, and 72.66% and 80.46% for indoor and outdoor CWs, respectively.
adapted from [53]	BTEX.	Upward flow in the pilot system. Sand/gravel CW	Successful.	Cumulative mass removal approached 80% for benzene and 88% for total BTEX.
adapted from [54]	BTEX.	Constructed wetland	Successful.	90% of BTEX removed.
adapted from [55]	12 organics including BTEX.	Hydroponic system	Successful.	BTEX compounds were translocated, and uptake was greatest for benzene.
adapted from [56]	BTEX	Bioremediation	Successful.	BTEX removal was high under WT dynamic conditions

## 11. Conclusions

This review paper focuses on the exploitation of microbes and plants as bioremediation tools to degrade organic contaminants in soil–water systems, which are lacking in a more comprehensive manner in the literature. This paper covers details of NAPL pollution and toxicity profiles of various BTEX contaminants, their fate, degradation and detoxification by microbes and plants or a consortium and associated challenges. Technological solutions such as duplex-constructed wetlands and/or integrated treatment wetlands can be a cost-effective solution for the hydrocarbon-contaminated soil–water system. Because many LNAPL compounds coexist with each other in the subsurface environment, further interactions of compounds need to be analysed for further expediency of biodegradation.

## Data Availability

All data is available with the corresponding author, will be shared on request.
